# Somatosensory Impairment and Chronic Pain Following Stroke: An Observational Study

**DOI:** 10.3390/ijerph20020906

**Published:** 2023-01-04

**Authors:** Brendon S. Haslam, David S. Butler, Anthony S. Kim, Leeanne M. Carey

**Affiliations:** 1 Occupational Therapy, School of Allied Health, Human Services and Sport, La Trobe University, Melbourne 3086, Australia; 2 Neurorehabilitation and Recovery, Florey Institute of Neuroscience and Mental Health, University of Melbourne, Melbourne 3010, Australia; 3 IMPACT in Health, University of South Australia, Kaurna Country, Adelaide 5001, Australia; 4 Neuro-Orthopaedic Institute, Adelaide 5000, Australia; 5 Weil Institute of Neurosciences, Department of Neurology, University of California, San Francisco, CA 94143, USA

**Keywords:** pain, stroke, chronic pain, sensation

## Abstract

Background: Chronic pain and somatosensory impairment are common following a stroke. It is possible that an interaction exists between pain and somatosensory impairment and that a change in one may influence the other. We therefore investigated the presence of chronic pain and self-reported altered somatosensory ability in individuals with stroke, aiming to determine if chronic pain is more common in stroke survivors with somatosensory impairment than in those without. Methods: Stroke survivors were invited to complete an online survey that included demographics, details of the stroke, presence of chronic pain, and any perceived changes in body sensations post-stroke. Results: Survivors of stroke (n = 489) completed the survey with 308 indicating that they experienced chronic pain and 368 reporting perceived changes in somatosensory function. Individuals with strokes who reported altered somatosensory ability were more likely to experience chronic pain than those who did not (OR = 1.697; 95% CI 1.585, 2.446). Further, this difference was observed for all categories of sensory function that were surveyed (detection of light touch, body position, discrimination of surfaces and temperature, and haptic object recognition). Conclusions: The results point to a new characteristic of chronic pain in strokes, regardless of nature or region of the pain experienced, and raises the potential of somatosensory impairment being a rehabilitation target to improve pain-related outcomes for stroke survivors.

## 1. Introduction

Following heart disease, a stroke is the most common cause of death and a leading cause of disability [1,2]. Due to recent improvements in medical management, there has been a substantial decrease in stroke mortality. This has resulted in an increased number of stroke survivors [3] and a subsequent increase in the burden of disability in the community. Individuals with strokes experience more difficulties participating in work and leisure activities than those without [4].

A common complication of strokes is the development of chronic pain. A prevalence of chronic pain in stroke populations is often reported as 40–65% [5,6]. This is more than twice that of the general population where it is commonly reported as 19–30% [7,8,9]. Survivors of stroke with chronic pain experience additional difficulties with cognitive function and the performance of physical activity than those without pain [10,11]. They also have higher rates of depression, fatigue, and anxiety [10,11]. Current guidelines [12,13] for stroke highlight the limited evidence for therapies targeting chronic pain in the stroke population.

Individuals with stroke who experience chronic pain are often classified into diagnostic groups, such as central post-stroke pain and post-stroke shoulder pain. Central post-stroke pain occurs in 7–8% of stroke survivors [14,15]. It is described as a central neuropathic pain condition due to a lesion of the central somatosensory nervous system [16] and is associated with sensory abnormalities in the body parts that correspond to the area of brain injury and an experience of pain attributed to the same body part. Interventions that have been studied and are currently recommended for neuropathic pain conditions, such as central post-stroke pain, are predominantly medication-based and associated with only modest effects [17,18]. Recognizing the need to improve the management of central post-stroke pain and to explore options other than medications, proposed recommendations have been made for the exploration of sensory-based rehabilitation approaches, and the measurement of potential effect of novel approaches targeting possible mechanisms involved in central post-stroke pain [19]. Post-stroke shoulder pain occurs more frequently, reported as being experienced by approximately one third of individuals with stroke [20,21]. Post-stroke shoulder pain has traditionally been attributed to musculoskeletal structural causes such as glenohumeral subluxation, impingement, tears of the rotator cuff complex, and joint capsulitis [22], i.e., being of more nociceptive in origin. However, there is a lack of supporting evidence for approaches targeting these, as reflected by current clinical guidelines for stroke [12,13,23]. A relationship between altered somatosensory processing and post-stroke shoulder pain has been observed [24,25], although the impact of somatosensory impairments on post-stroke shoulder pain has been questioned [26]. To the authors’ knowledge, there is currently no published literature regarding the use of sensory-based treatments for post-stroke shoulder pain.

Recent studies have demonstrated a relationship between altered somatosensory discrimination abilities and chronic pain in a range of other pain states that are not associated with central lesions (e.g., non-specific low back pain) [27,28], complex regional pain syndrome (CRPS) [29], and chronic neck pain [30]. Further, it is considered that individuals with chronic pain and observed changes in sensory function (such as tactile acuity) also exhibit disturbances of body image [31]. Novel interventions addressing somatosensory dysfunction, in particular discrimination training of sensory features, such as tactile acuity, have achieved significant reductions of pain levels in several complex chronic pain states including non-specific low back pain [32], phantom limb pain [33], and CRPS [34].

The presence of somatosensory impairment in individuals with stroke who may experience chronic pain more broadly (i.e., that cannot be classified into either central post-stroke pain or post-stroke shoulder pain) has not been investigated. We aimed to identify if there was a relationship between chronic pain and self-reported somatosensory impairment following stroke, and if so, if there were particular somatosensory features that were more prominent. If a relationship exists, interventions targeting the somatosensory system could be indicated in the treatment of chronic pain in stroke survivors.

## 2. Materials and Methods

### 2.1. Design

An anonymous online survey was designed for the purpose of this study for individuals who had experienced a stroke or multiple strokes. The survey sought age and gender data, details of stroke, whether they had chronic pain (i.e., pain that had been present for more than three months), and if they reported any post-stroke somatosensory impairment. Participants were asked “Have you had any changes in ability to feel things since the stroke? YES/NO”. If the individuals indicated “YES”, they were then invited to answer further questions regarding the nature of their perceived somatosensory changes. These questions were designed to highlight any perceived changes in superficial touch detection and discrimination, proprioception (i.e., ability to sense body positions), and changes in haptic object recognition (which requires the use of a range of exploratory procedures for somatosensory discrimination [35]). The survey included opportunities for participants to make any additional comments throughout. A copy of the survey questions related to the presence of altered sensation and pain have been included (Appendix A). Prior to the commencement of recruitment, pilot trials of the survey were performed by individuals with strokes, and survey completion was found to take approximately 15 min to complete. The study was approved by the Human Research Ethics Committee of the University of Melbourne (ID 1340670), the Human Ethics Committee of La Trobe University, and the Institutional Review Board of the University of California, San Francisco. Participants were recruited through multiple means including flyers, newsletters, website listings, social media links, and via an established research register for stroke survivors who had indicated a willingness to participate in research activities. Data were collected between October 2015 and October 2018. A required sample size of 426 was calculated a priori for 95% power to observe a small effect (0.2) or larger at a significance level of 0.008.

This manuscript conforms to STROBE (Strengthening the Reporting of Observational Studies in Epidemiology) guidelines [36].

### 2.2. Participants

Participants were included in the study if they reported experiencing a stroke a minimum of three months prior to completing the survey, were over eighteen years of age, and able to provide consent. Potential participants were excluded if they had been diagnosed with a neurological condition other than a stroke or were unable to read English.

### 2.3. Data Analysis

Following the application of the exclusion criteria, participant data was reviewed. Participants were allocated into groups according to the reported presence of somatosensory impairment or not for the primary analysis and were excluded if missing data made grouping not possible. To identify other potential factors that may be associated with chronic pain, the mean age of participants and chronicity of stroke were calculated and compared for those with and without chronic pain using the Student *t*-test. Potential differences in gender and the reported hemisphere of the lesion were compared using the chi-square test. Similarly, age, chronicity of stroke, gender, and the reported hemisphere of the lesion were investigated for potential differences in those with and without the presence of perceived somatosensory impairment.

The primary analysis investigated presence of a significant relationship between the frequency of occurrent of chronic pain in those with and without self-reported sensory impairment using the chi-square test. A between-group difference was deemed significant at *p* < 0.05. Odds ratios with 95% confidence intervals were generated to determine the strength of the association if present. Secondary analyses investigated between-group differences according to the presence of the altered self-reported sensation in those with and without chronic pain, in addition to the observed frequency of the varying somatosensory impairment features, with differences deemed significant at *p* < 0.008, following a correction for multiple comparisons. Odds ratios with 95% confidence intervals were again generated to determine the strength of the association if present.

## 3. Results

### 3.1. Flow of Participants through the Study

A total of 533 individuals with chronic stroke from 36 countries provided consent and participated in the survey, with the majority being from the United States of America (315) and Australia (123). Twenty-six were excluded due to the presence of other neurological conditions and eighteen due to incomplete data that did not permit group allocation. Data analysis was carried out on 489 participants (368 with reported somatosensory impairment, 121 who reported no impairment).

### 3.2. Frequencies of Impairment and Characteristics of Subgroups

The mean age, gender distribution, chronicity of stroke, and reported hemisphere of the lesion for those with and without chronic pain are shown in Table 1. Differences in the reporting of chronic pain by gender were observed. There were no differences between groups in age, duration of post-stroke, or reported hemisphere of lesion. The mean duration post-stroke was 7–8 years, reflecting a chronic stroke population. No comments were made by participants to indicate that they did not understand the questions.

The frequency of presence of self-reported somatosensory impairment or the absence of impairment and associated characteristics are presented in Table 2. There were no differences detected for age, gender distributions, chronicity of stroke, nor reported hemisphere of the lesion.

### 3.3. Reported Chronic Pain in Stroke Survivors with Somatosensory Impairment

Group comparisons were performed based on the presence of self-reported somatosensory impairment. Individuals with stroke who reported somatosensory impairment experienced higher rates (70%) of chronic pain than those without reported somatosensory impairment (41%) (OR = 1.697; 95% CI 1.585, 2.446). Observed frequencies for the presence of chronic pain according to somatosensory impairment are reported in Table 3.

### 3.4. Self-Reported Somatosensory Impairment with Chronic Pain

Individuals with strokes who experienced chronic pain reported higher rates (84%) of changes in their ability to feel things since their stroke than those without chronic pain (61%) (OR = 1.378; 95% CI 1.032, 1.840).

### 3.5. Features of Self-Reported Somatosensory Impairment and Chronic pain

Individuals with strokes who experienced chronic pain reported significantly higher rates of somatosensory impairment in all sensory domains asked by the survey—light touch (OR = 1.638; 95% CI 1.159, 2.313), body position (OR = 1.816; 95% CI 1.273, 2.591), tactile discrimination of surface textures (OR = 1.816; 95%CI 1.273, 2.591), temperature discrimination (OR = 1.464; 95% CI 1.018, 2.104), and the ability to recognise objects by touch alone (OR = 1.501; 95% CI 1.068, 2.110)—compared to those without chronic pain. Differences in being able to clearly feel light touch, the position of body parts, differences in the texture of surfaces, and recognise objects by touch alone were reported equally by those with chronic pain (54–55%) while differences in hot or cold appreciation were less frequent (44%). The observed frequencies for each sensory feature (domain) are reported in Table 4.

## 4. Discussion

A significant relationship was found between self-reported somatosensory impairment and the presence of chronic pain in stroke survivors at least three months post-stroke. This relationship is unlikely to be explained by individual participant characteristics such as age, chronicity of stroke or reported hemisphere of lesion, as these factors did not differ between subgroups with and without pain nor subgroups with and without self-reported somatosensory impairment. While participants who identified as female reported higher rates of chronic pain, gender distributions did not differ for those with and without somatosensory impairment and so is unlikely to explain the relationship between reported pain and altered somatosensory function. Our finding of more females identifying as having chronic pain in a population of stroke survivors is unsurprising, as this is commonly reported in many chronic pain states [8,9,37].

To the best of our knowledge, this study is the first to investigate if a relationship exists in individuals with stroke, regardless of the nature or region of chronic pain. Two commonly reported pain conditions experienced post-stroke (central post-stroke pain and post-stroke shoulder pain) have previously been associated with somatosensory impairment. Post-stroke shoulder pain in the first six months after a stroke has been associated with somatosensory loss to both innocuous and noxious stimuli on the affected side and sensitization to cold and multimodal stimuli [38], supporting the notion that central sensitization may play a role in the development and/or maintenance of post-stroke shoulder pain. Studies investigating post-stroke shoulder pain [26,39] have reported on sensory detection thresholds as measured by techniques such as quantitative sensory testing (QST), but significant variability in QST measurements have been recorded, and the need for a test protocol with reduced variability has been identified [39].

One study that compared stroke survivors with and without shoulder pain found no significant difference between the groups for the prevalence of somatosensory impairment when measured by QST and questioned the impact of somatosensory impairments on shoulder pain [26]. Further examination of participant data in this study who did not have shoulder pain however found that forty percent of those reported pain in other regions of their body. Our study did not localize to region of pain or somatosensory impairment, instead focusing on participants self-reporting of sensory function.

The response rate of 75% of our survey sample indicating an altered ability to feel things is within previously reported ranges of somatosensory impairment following stroke [40,41]. The survey questions provided opportunities to focus on perceived somatosensory function, in particular discrimination abilities (e.g., *“are you sometimes unable to tell the difference between surfaces…”*; *“are you sometimes unable to tell the difference between hot and cold…”*), rather than detection thresholds as used in QST studies. This enabled the exploration of perceived somatosensory changes in touch detection (light touch) and tactile discrimination, in addition to discrimination of limb position sense, and discrimination of object recognition that involves the use of multiple exploratory procedures (e.g., tactile discrimination, contour following, pressure). We found a strong relationship between chronic pain and somatosensory impairment in individuals with stroke. Further, this association was evident for the different features of somatosensory impairment reported, with similar frequencies of reported impairment for most features of somatosensation asked in the survey. Consistent with previous findings for individuals with stroke [40], an altered temperature discrimination ability was found to be less common than features of light touch detection, tactile discrimination, limb position sense, and haptic object recognition. In this sample, survivors of strokes often experienced impairment of multiple features of somatosensation, suggesting that there is not a distinct somatosensory feature that is most commonly associated with the experience of chronic pain.

The development of chronic pain post-stroke is generally considered to occur over a period of months following stroke [42,43,44]. This development of pain over time is suggestive that adaptive, neuroplastic processes occurring following a stroke may contribute towards the pain experience, rather than pain being solely due to the neurological lesion or a musculoskeletal injury. If this is the case, then chronic pain following stroke may be considered to have combinations of neuropathic, nociceptive, and nociplastic characteristics in line with current definitions described by the International Association for the Study of Pain [45]. Nociplastic characteristics imply that adaptive processes, such as central sensitization, may have occurred that contribute to the development and maintenance of a pain experience. Further adaptations are also possible, which may be perceived to be either positive (towards a reduction of pain) or maladaptive (towards a further increase in pain).

Discrimination training of upper limb somatosensory function in individuals with stroke has been demonstrated to be effective in improving touch detection, somatosensory discrimination, and the functional use of the affected limb [46,47], but its role in chronic pain for stroke survivors has not been specifically investigated. The training of tactile acuity through methods of discrimination and localization has demonstrated positive results in pain reduction for a variety of complex chronic non-stroke pain conditions where impaired tactile acuity and altered body image have been demonstrated to be present, including CRPS [34], non-specific low back pain [32], and phantom limb pain [33]. The potential mechanisms behind this have been proposed to be due to repeated exposure to stimuli that could be perceived as threatening and cortical reorganization that is in response to discrimination training [34].

Individuals with stroke commonly experience CRPS [48] and persistent pain previously thought to be of musculoskeletal origin [49] with nociceptive characteristics similar to those of persistent non-specific low back pain. The findings of this study indicate that chronic pain is more likely to be experienced by individuals with chronic stroke who report altered ability in their sensation. This is in addition to those diagnosed with central post-stroke pain [14,15]. This finding not only provides support for an earlier recommendation for the use of interventions targeting the sensory system (e.g., somatosensory retraining) to be explored in central post-stroke pain [19], but also support for its use more broadly for individuals with stroke who experience chronic pain, even in pain experiences that may previously have been considered to be of musculoskeletal or typically nociceptive in origin, recognizing the mixed nature of chronic pain. It is reasonable to propose that improving somatosensory abilities and functional use of the vulnerable body part through sensory retraining (in particular discrimination training) will act to enhance the perceived robustness of the individuals body image and reduce the perceived threat of external stimuli (e.g., interaction via touch with other objects or people) and internal stimuli (e.g., proprioceptive processing of movement) through repeated positive exposure. Increasing the engagement and functional use of the upper limb in meaningful activities that require processing of somatosensory information to enable successful task performance will continue to drive opportunities for repeated exposure and cortical change, further reducing the need for protective strategies such as pain.

Interventions other than discrimination training have been explored to address the issue of somatosensory impairment, such as transcranial magnetic stimulation in chronic stroke [50], and transcranial direct current stimulation in subacute stroke [51], showing a potential benefit. It has been identified, however, that further trials with longer follow-up periods are needed [52], and the use of non-invasive brain stimulation for somatosensory impairment is currently not recommended in clinical guidelines [12].

There are several limitations to this study. As the study was performed online and in English, participants were required to have internet access, adequate technology skills, and be proficient in the English language. Unfortunately, there is currently no pain assessment measure available for individuals with aphasia that is suitable for an online study of this type [53], and as such, stroke survivors with aphasia will have found it difficult to participate. This study reported on somatosensory function purely as reported by the participants rather than objective physical measurement techniques. Physical assessments of somatosensation for stroke survivors exist such as quantitative sensory testing [39], in addition to the broad Nottingham Sensory Assessment [54] or more specific and quantitative Tactile Discrimination Test [55], Wrist Position Sense Test [56], and the functional Tactile Object Recognition Test [35]. Given that this was an exploratory study looking in principle to explore a possible relationship between somatosensation and chronic pain in stroke survivors, regardless of pain type, a survey was deemed the most effective method to ensure a large sample size and range of pain experiences. In addition, if the individual with a stroke perceives that their sensation is impaired (as indicated by self-reporting), this could then contribute to a feeling of vulnerability of the body part affected, and thus be perceived as being more susceptible to damage. This could be an important factor in the perceived need of the individual to evoke a protective strategy such as pain due to a reduction of perceived body safety [57], resulting in further reductions in use and exposure to stimuli of the affected body part. Further studies combining subjective reporting and objective testing of somatosensation function (including discrimination abilities) in individuals with strokes who experience chronic pain would be beneficial to further investigate these findings and explore the potential therapeutic implications.

In using the recruitment strategy outlined, we were unable to determine the response rate for this study. We were able to determine a completion rate, however, and our non-completion rate of four percent compares favorably to expected rates for non-incentive studies that are conducted online, which are often associated with incomplete data [58].

## 5. Conclusions

The findings from this study provide support for an association between perceived (self-reported) somatosensory impairment and chronic pain post-stroke regardless of pain type (e.g., neuropathic, nociceptive/musculoskeletal) or region (e.g., shoulder). This new finding contributes to the growing understanding of chronic pain in strokes and provides clinicians with insights into the relationship that exists with stroke between pain and somatosensation. It is hoped that this new knowledge will contribute to the early identification and exploration of existing strategies targeting somatosensation and chronic pain.

## Figures and Tables

**Table 1:** Age, gender, stroke chronicity and reported hemisphere of lesion reported within samples with and without chronic pain.

	Chronic Pain (n = 308)	No Pain (n = 181)	*p* Value
Age: years (mean, SD) Gender: female (number, percentage) Duration post-stroke: years (mean, SD) Reported hemisphere of lesion ● Right ● Left ● Both ● Unknown	58 (12)	59 (12)	*p* = 0.265 ^a^
	176 (57%)	77 (43%)	*p* = 0.005 ^b^
	7 (7) 148 (48%) 109 (35%) 23 (7%) 28 (9%)	8 (7) 85 (47%) 67 (37%) 9 (5%) 20 (11%)	*p* = 0.386 ^a^ *p* = 0.816 ^b^ *p* = 0.717 ^b^ *p* = 0.281 ^b^

^a^ Based on Student *t*-test; ^b^ Based on Chi-Square test.

**Table 2:** Age, gender, stroke chronicity and reported hemisphere of lesion reported within samples with and without perceived presence of somatosensory impairment.

	Self-Reported Somatosensory Impairment (n = 368)	Self-Reported No Somatosensory Impairment (n = 121)	*p* Value
Age: years (mean, SD) Gender: female (number, percentage) Duration post-stroke: years (mean, SD) Reported hemisphere of lesion ● Right ● Left ● Both ● Unknown	57 (12)	58 (12)	*p* = 0.353 ^a^
	186 (48%)	66 (55%)	*p* = 0.445 ^b^
	7 (7) 179 (49%) 132 (36%) 24 (7%) 33 (9%)	6 (6) 54 (45%) 44 (36%) 8 (7%) 15 (12%)	*p* = 0.091 ^a^ *p* = 0.443 ^b^ *p* = 0.922 ^b^ *p* = 0.972 ^b^

^a^ Based on Student *t*-test; ^b^ Based on Chi-Square test.

**Table 3:** Relationship between reported chronic pain and self-reported somatosensory impairment after stroke, with confidence intervals (95%) indicated.

	Self-Reported Somatosensory Impairment (n = 368)	Self-Reported No somatosensory Impairment (n = 121)	*p* Value
Experience of chronic pain	258 (70%) (65–75)	50 (41%) (33–50)	*p* < 0.001 ^b^

^b^ Based on Chi-Square test.

**Table 4:** Relationship between reported presence and/or features of somatosensory impairment and chronic pain after stroke, with confidence intervals (95%) indicated.

	Chronic Pain (n = 308)	No Pain (n = 181)	*p* Value
Have you had any changes in your ability to feel things since the stroke?	258 (84%) (80–88)	110 (61%) (54–68)	*p* < 0.001 ^b^
Are you sometimes unable to clearly feel being lightly touched?	170 (55%) (50–61)	61 (34%) (27–41)	*p* < 0.001 ^b^
Are you sometimes unsure of the position of any of your body parts?	169 (55%) (49–60)	60 (33%) (26–40)	*p* < 0.001 ^b^
Are you sometimes unable to tell the differences between surfaces (e.g., rough/smooth) with your hands/fingers?	170 (55%) (50–61)	55 (30%) (24–37)	*p* < 0.001 ^b^
Are you sometimes unable to tell the difference between hot and hold with your hands/fingers?	137 (44%) (39–50)	55 (30%) (24–37)	*p* = 0.002 ^b^
Are you sometimes unable to recognize objects by touch alone with your hands?	166 (54%) (48–59)	65 (36%) (29–43)	*p* < 0.001 ^b^

^b^ Based on Chi-Square test.

## Data Availability

The data presented in this study are available on request from the corresponding author. The data are not publicly available due to planned further analyses.

## Appendix A

Survey questions for somatosensation and pain

1. Changes to your sensation
  - Have you had any changes in your ability to feel things since the stroke? YES/NO
  - If yes, please continue:
    - Are you sometimes unable to clearly feel being lightly touched? YES/NO
    - Are you sometimes unsure of the position of any of your body parts? YES/NO
    - Are you sometimes unable to tell the difference between surfaces (e.g., rough/smooth) with your hands/fingers? YES/NO
    - Are you sometimes unable to tell the difference between hot and cold with your hands/fingers? YES/NO
    - Are you sometimes unable to recognize objects by touch alone with your hands? YES/NO
2. Pain
  - Have you experienced persistent pain over the past three months that has made you do something for it? (e.g., take a tablet, change behaviors, see a health professional) YES/NO
